# Detection of a Novel *DSPP* Mutation by NGS in a Population Isolate in Madagascar

**DOI:** 10.3389/fphys.2016.00070

**Published:** 2016-03-02

**Authors:** Agnès Bloch-Zupan, Mathilde Huckert, Corinne Stoetzel, Julia Meyer, Véronique Geoffroy, Rabisoa W. Razafindrakoto, Saholy N. Ralison, Jean-Claude Randrianaivo, Georgette Ralison, Rija O. Andriamasinoro, Rija H. Ramanampamaharana, Solofomanantsoa E. Randrianazary, Louise H. Ralimanana, Béatrice Richard, Philippe Gorry, Marie-Cécile Manière, Jeanne A. Rasoamananjara, Simone Rakoto Alson, Hélène Dollfus

**Affiliations:** ^1^Faculté de Chirurgie Dentaire, Université de Strasbourg Strasbourg, France; ^2^Centre de Référence des Manifestations Odontologiques des Maladies Rares, Hôpitaux Universitaires de Strasbourg, Pôle de Médecine et Chirurgie Bucco-dentaires Hôpital Civil Strasbourg, France; ^3^Centre National de la Recherche Scientifique-UMR7104, Institut de Génétique et de Biologie Moléculaire et Cellulaire, Institut National de la Santé et de la Recherche Médicale U 964, Université de Strasbourg Illkirch, France; ^4^Laboratoire de Génétique Médicale, Faculté de Médecine, Institut National de la Santé et de la Recherche Médicale U 1112, Université de Strasbourg Strasbourg, France; ^5^Institut d'Odonto-Stomatologie Tropicale de Madagascar, Université de Mahajanga Mahajanga, Madagascar; ^6^UFR Odontologie de Lyon, Université Claude Bernard Lyon, France; ^7^Research Unit of Theoretical & Applied Economics, GREThA (UMR Centre National de la Recherche Scientifique 5113), Université de Bordeaux Pessac, France

**Keywords:** rare disease, dentinogenesis imperfecta, dental anomalies, dentin, mutations, NGS, human

## Abstract

A large family from a small village in Madagascar, Antanetilava, is known to present with colored teeth. Through previous collaboration and 4 successive visits in 1994, 2004, 2005, and 2012, we provided dental care to the inhabitants and diagnosed dentinogenesis imperfecta. Recently, using whole exome sequencing we confirmed the clinical diagnosis by identifying a novel single nucleotide deletion in exon 5 of *DSPP*. This paper underlines the necessity of long run research, the importance of international and interpersonal collaborations as well as the major contribution of next generation sequencing tools in the genetic diagnosis of rare oro-dental anomalies. This study is registered in ClinicalTrials (https://clinicaltrials.gov) under the number NCT02397824.

## Introduction

Dentinogenesis imperfecta (DI) belongs to a group of rare genetic diseases affecting the formation/mineralization of tooth dentin and is transmitted, as recorded so far, in an autosomal dominant manner (Barron et al., 2008). A dominant negative effect of a modified dentin sialophosphoprotein (DSPP) has been suggested as the pathogenic mechanism underlying DI (Wang et al., 2011).

These disorders exist either in isolation, with clinical manifestations limited to the oral cavity and are named dentinogenesis imperfecta type II (DGI-II) or hereditary opalescent dentin (OMIM #125490) also called dentinogenesis imperfecta 1 (DGI-1) or Capdepont teeth, and dentinogenesis imperfecta type III (DGI-III; OMIM # 125500) (Shields et al., 1973; Kim and Simmer, 2007; Barron et al., 2008; de la Dure-Molla et al., 2015). They can be associated with other symptoms like progressive sensorineural hearing loss (OMIM # 605594) (Xiao et al., 2001) or encountered in syndromes like osteogenesis imperfecta and Goldblatt syndrome for example in which bone defects (a tissue similar to dentin) are key features of the clinical synopsis (Bloch-Zupan et al., 2012; Bloch-Zupan, 2014). Mutations described so far occur in one single gene *DSPP* (4q21.3), belonging to the SIBLINGs family and encoding three major non-collagenous dentin matrix proteins- dentin sialoprotein (DSP), dentin glycoprotein (DGP) and dentin phosphoprotein (DPP) (Zhang et al., 2001; MacDougall, 2003; Kim et al., 2005; Lee et al., 2008, 2009, 2011a, 2013).

In this paper, we focus on a large family from a small village in Madagascar, Antanetilava, known to present with colored teeth. The aim of the study is, through phenotyping and genotyping, to unravel the diagnosis and genetic origin of this rare familial condition. Through previous collaboration (1996) and successive visits in 2004, 2005 and 2012, we provided dental care to the inhabitants and linked the discoloration diagnosis to dentinogenesis imperfecta. Recently, using whole exome sequencing we confirmed this diagnosis by identifying a novel *DSPP* mutation segregating with the disease in this family.

## Materials and methods

### Patients

In 1996 (Razafindrakoto et al., 1996), the Strasbourg Faculty of Dentistry and the INSERM_U424 (JV Ruch) were contacted by colleagues from l'IOSTM (Institut d'Odonto-Stomatologie Tropicale de Madagascar) of Mahajanga University to help in disseminating scientific data related to a specific family originating from the small isolated village of Antanetilava (18°58′42.2″S 47°14′01.9″E), in the middle of the luxuriant tropical forest, in the Toamasina province, located 40 km North-West from the city of Toamasina. This region on the East coast of Madagascar is known for its hot and humid climate. It is a rural area devoted essentially to agriculture where rice, manioc, potatoes, banana and yam are cultivated. Colleagues visited Antanetilava in 1994, when a total of 110 inhabitants lived there. Problems related to inherited tooth anomalies in the population of Antanetilava had been previously noticed and the aim of this first visit was to determine what the tooth defects were, to follow the disorder in the families and to estimate its frequency.

At that time 50 people (28 females and 22 males) belonging to 22 of 26 households in the village were examined. Eleven individuals (22%, 6 females and 5 males) presented with this colored teeth anomaly affecting both the primary and permanent dentition. Clinical examination revealed brown to blue-gray discoloration of the crowns. Severe attrition due to early enamel chipping was visible. A clinical diagnosis of dentinogenesis imperfecta type II was proposed. Radiographic examination was possible for one 23 year-old patient in the nearest local hospital of Toamasina. Progressive pulp chamber obliterations as well as absent root canals were noticed, confirming the diagnosis. A first pedigree was drawn and demonstrated that the genetic disease affected 5 generations and 46.7% of the family members.

After a 2004 preparatory mission, JM returned to Mahajanga and visited Antanetilava in 2005 after a difficult journey consisting of 6 h of bus, 8 h of bush taxi, 1 dugout canoe river crossing and a further hour of walking.

Twenty seven participants (25 affected) and 2 non-affected family members were examined during this 2005 visit. Affected and unaffected family members gave written informed consent and, the study was approved by the village council. The orodental phenotypes were documented using the D[4]/phenodent registry: a Diagnosing Dental Defects Database (see www.phenodent.org, to access assessment form). This registry allows the standardization of data collection and assists in orodental phenotyping. It also facilitates providing clinical care to patients, a basis for genotype/orodental phenotype correlations, and sharing of data and clinical material between clinicians. D[4]/phenodent registry is approved by CNIL (French National commission for informatics and liberty) under the following number 908416.

This clinical study has been registered in Clinical trials (https://clinicaltrials.gov) under the number NCT02397824 and is registered by the French Ministère de l'enseignement supérieur et de la recherche, DGRI/Cellule de bioéthique (bioethics committee) under DC-2012-1677. It was acknowledged by the CPP (person protection committee) Est IV on the 11/12/2012.

### Mutation analysis

JM collected DNA samples using Whatman FTA cards and Oragene® DNA kits.

Genomic DNA was isolated from the saliva of 14 family members (9 affected and 5 unaffected), during the 2012 mission, using the prepIT-L2P OG-250 Oragene® DNA kit (DNA Genotek Inc., Ontario, Canada) according to standard protocols.

A few attempts of direct *DSPP* Sanger sequencing were unsuccessful.

We performed whole-exome sequencing (IntegraGen, Evry, France) for five affected patients (III.15, III.32, IV.26, IV.57, and IV.65) and one healthy individual (IV.22). Exons of DNA samples were captured using in-solution enrichment methodology (SureSelect Human All Exon Kits Version 3, Agilent, Massy, France) with the company's biotinylated oligonucleotide probe library (Human All Exon v5+UTR—75 Mb, Agilent). The genomic DNA was then sequenced on a sequencer as paired-end 2X75 base pair reads (Illumina HISEQ2000, Illumina, San Diego, USA) resulting in an average coverage of 200X. Image analysis and base calling was performed using Real Time Analysis (RTA) Pipeline version 1.9 with default parameters (Illumina). The bioinfomatic analysis of sequencing raw data was based on the pipeline provided by the company (CASAVA 1.8, Illumina and finally detects from 80965 to 82263 variants (SNPs and Indels) per proband (Table 1). Annotation, ranking, and filtering of genetic variants were performed with the VaRank program (Geoffroy et al., 2015). Very stringent criteria were used for excluding non-pathogenic variants, in particular: (1) variants represented with an allele frequency of more than 1% in dbSNP 138, the EXAC database or the NHLBI Exome Sequencing Project Exome Variant Server (EVS), (2) variants found at the homozygous state or more than once at the heterozygous state in 48 control exomes, (3) variants in the 5′ or 3′ UTR, (4) variants with intronic locations and no prediction of local splice effect, and (5) synonymous variants without prediction of local splice effect.

**Table 1:** **Summary of the exome sequencing results**.

**Individuals**	**III.15**		**III.32**		**IV.22**		**IV.26**		**IV.57**		**IV.65**	
**Type of sequence variant**	**SNV**	**Indel**	**SNV**	**Indel**	**SNV**	**Indel**	**SNV**	**Indel**	**SNV**	**Indel**	**SNV**	**Indel**
Total number of variants	72429	9001	72273	8692	72638	8960	73233	9030	72480	8908	72212	8970
After exclusion of non-pathogenic variants (as determined from the ClinicalSignificance field in dbSNP) validated by at least 2 methods in dbSNP (as determined from the “Validation Status” field)	8767	4955	8617	4658	8745	4898	8758	4940	8576	4812	8564	4852
After exclusion of variants with an allele frequency > 1% (extracted from the EXAC database, the Exome Variant Server and the dbSNP database)	5734	3122	5653	3035	5792	3117	5703	3162	5675	3106	5648	3091
After exclusion of variants found in the homozygous state or more than once in the heterozygous state in 70 control exomes	1972	843	1998	910	2041	888	1990	901	2115	923	2234	884
After exclusion of 5′UTR, 3′UTR, downstream, upstream and intron locations without local splice effect prediction (from the “localSpliceEffect” field of Alamut-Batch)	767	123	729	132	757	119	706	120	731	122	780	119
After exclusion of synonymous variants without local splice effect prediction (from the “localSpliceEffect” field of Alamut-Batch)	571	123	563	132	571	119	520	120	569	122	592	119
Selection of variants consistent with recessive transmission	0 compound heterozygous											
	0 homozygous variants											
Selection of variants consistent with dominant transmission.	4 heterozygous variants (in the *DSPP, ABHD14A-ACY1, ABHD14A, HERC6* and *THAP9* genes)											

Sanger sequencing (GATC Biotech, Applied Biosystems ABI 3730xl™, Konstanz, Germany) was used to validate the mutations and verify segregation using the following primers.

Specific forward (F) and reverse (R) primers were designed to amplify the *DSPP* exon 5 region containing the mutation: DSPP-F (GTGACAGCAGCAATAGCAGTGATA) and DSPP-R (TCACTGGTTGAGTGGTTACTGTC) (expected product size of 376 bp (base pair). PCR amplifications were performed in a final volume of 50 μl containing 0.2 μM forward and reverse primers, 0.2 mM dNTPs, 1X GoTaq reaction buffer containing 1.5 mM MgCl2, 1.25 unit of GoTaq DNA polymerase (Promega), 50 ng of template DNA and 3% DMSO. Amplifications were performed for 40 cycles, each consisting of 30s denaturation at 94°C, 30s annealing at 64.9°C and 17s elongation at 72°C.

## Results

### Clinical phenotype

**Figure 1:**
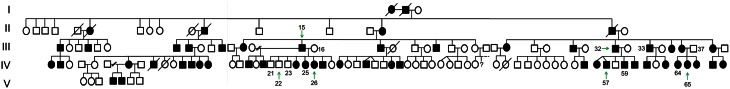
**Family pedigree**. A large pedigree, spanning 5-generations with 137 individuals, of which 55 are affected, is showing a dominant inheritance pattern. Arrows point to individuals whose exomes were sequenced (affected: III.15, III.32, IV.26, IV.57, IV.65; non-affected: IV.22). Sanger sequencing was performed for the following subjects: affected (III.15, III.32, III.33, IV.25, IV.26, IV.57, IV.59, IV.64, IV.65), non-affected (III.16, III.37, IV.21, IV.22, IV.23).

The family history and pedigree, as established in 1996, was updated. It then included 137 individuals spanning 5 generations (Figure 1) and 4 related families. 55 individuals, 31 males and 24 females, were reported as affected and presenting with DI corresponding to a 40.1% prevalence of the disease within this population (1/2 in generation I, 2/3 in generation II, 4/13 or 30.8% in generation III, 16/25 (64%) in IV, 30/64 (46.9%) in V and 2/7 (28.6%) in VI).

Medical history was collected from the 25 affected of the 27 examined persons. Patients reported only infectious episodes like malaria, measles, and fever. Some affected individuals (3) presented a triangular face shape or a facial asymmetry. Most of affected persons (23) showed blue sclera. Disturbance of hearing was recorded for 5 affected individuals. 9 patients presented articular distortions or pain and 3 had nail dysplasia.

**Figure 2:**
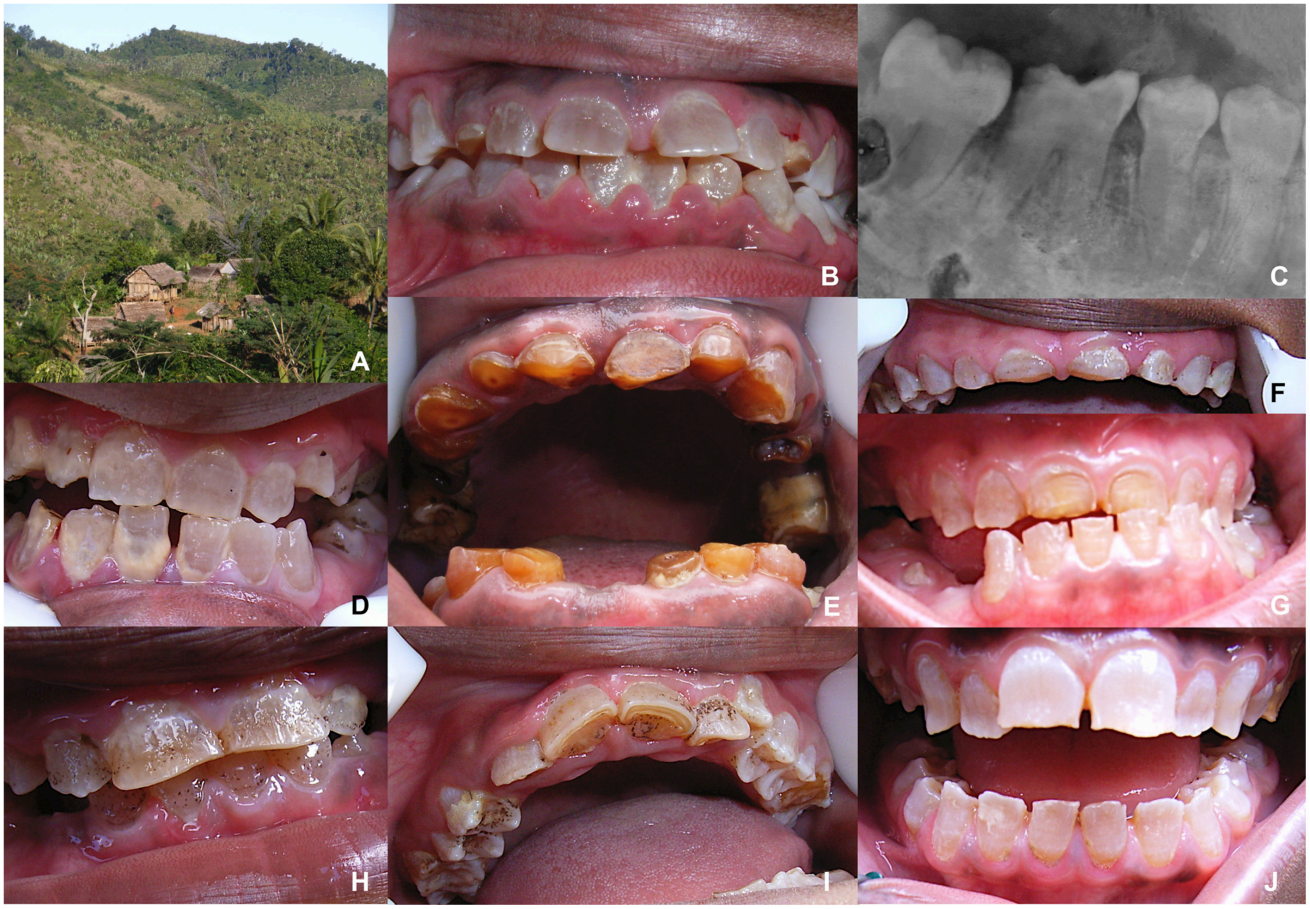
**Clinical description of the disease. (A)** The remote village of Antananarivo. **(B–J)** Inhabitants'dentition showing the typical features of dentinogenesis imperfecta with the gray-brown discolouration of the dentin clearly visible after enamel cleavage and progressive tooth wear. **(C)** On the retro-alveolar radiography of the lower right premolar/molar sector of individual **(B)**, cervical constriction, short roots and the disappearance of pulp spaces due to erratic dentin formation represent the characteristic hallmarks of dentinogenesis imperfecta. **(H–J)** In addition to dentin anomalies, hypoplastic enamel defects exist, with the presence of pits, striae and flattened buccal surfaces.

Dental history mentioned infections, early tooth mobility and loss and tooth extractions. Both the primary and permanent dentitions were affected. Teeth presented with the amber-gray color pathognomonic of heritable dentin defects (Figure 2). Some tooth shape/size anomalies were observed as scoop shaped incisors, absence of convex vestibular crown surface, flat aspect of crown occlusal surfaces and supernumerary cusps. Enamel, when visible, presented an irregular appearance. Tooth wear was considerable and was visible via the colored abnormal dentin after enamel shedding. Fifteen individuals (11 adults, 4 children) suffered from tooth mobility. Nine individuals experienced dental infections. A probable diagnosis of DI was made. Three affected patients benefitted from X-ray investigations through intraoral radiographs taken in a private practice in the Antananarivo town. These pictures showed complete pulp space obliteration and globular crowns with cervical constrictions (Figure 2) confirming the diagnosis.

### Genotype

Using VaRank, to annotate rank, and filter the genetic variants, we identified, amongst 80965–82263 variants (SNPs and Indels) per proband, four candidate variants in five genes (*DSPP* [dentin sialophosphoprotein, OMIM: 125485], *ABHD14A-ACY1* [ABHD14A-ACY1 readthrough (NMD candidate)], *ABHD14A* [abhydrolase domain containing 14A], *HERC6* [HECT and RLD domain containing E3 ubiquitin protein ligase family member 6, OMIM: 609249] and *THAP9* [THAP domain containing 9, OMIM: 612537]) with heterozygous variants present only in the affected patients (Table 1). We then focused the subsequent study on the heterozygous variant in *DSPP*, a known causal gene for DI: a heterozygous deletion (c.3676del [p.Ser1226Alafs^*^88] [RefSeq NM_014208.3]). Mutation is absent from the 1000 genome, NHLBI EVS and ExAC databases.

**Figure 3:**
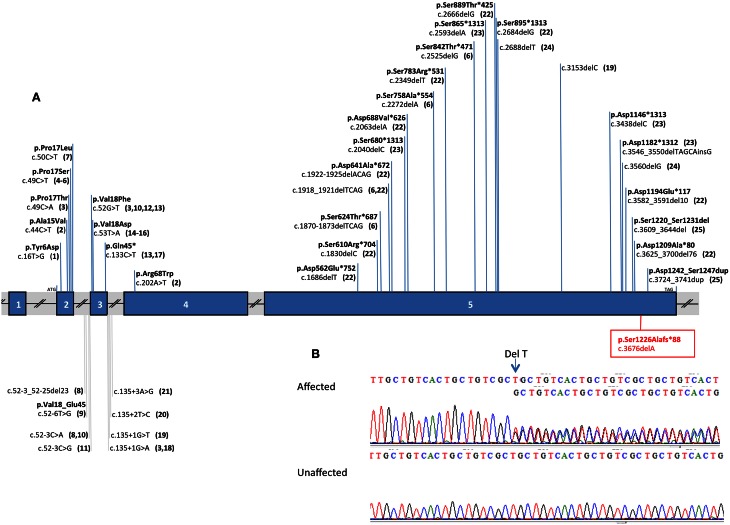
***DSPP* mutation analysis**. *DSPP* mutations are indicated against the gene structure (reference sequence: NM_014208.3). **(A)** Schematic representation of *DSPP*: this gene contains 5 exons (vertical blue hatches), the position of the start codon (ATG) and the stop codon (TAG) are indicated respectively in exon 2 and exon5. The known mutations in the *DSPP* gene are summarized against the gene structure and associated to a literature reference. The new mutation described in this paper is boxed and written in red. For example: the most 5′ *DSPP* mutation, near the initiation codon (ATG) is lying in exon 2 and described as a single nucleotide variant c.16T>G leading to the following amino acid changes in the protein p.Tyr6Asp and reported in the literature in quoted reference (Rajpar et al., 2002). **(B)** Electrophoregrams of a part of *DSPP* exon 5 showing the heterozygous mutation in an affected person and the normal sequence in an unaffected individual. The deletion of 1T is indicated with an arrow, this deletion creates a shift in the reading frame in position 3676 of the cDNA reference sequence, resulting in 2 superposed sequences. On the scheme the numbering corresponds to the following references: **1**. Rajpar et al. (2002); **2**. Malmgren et al. (2004); **3**. Xiao et al. (2001); **4**. Zhang et al. (2007) and Qu et al. (2009); **5**. Hart and Hart (2007); **6**. Mcknight et al. (2008a); **7**. Li et al. (2012) and Lee et al. (2013); **8**. Wang et al. (2009); **9**. Lee et al. (2008); **10**. Holappa et al. (2006); **11**. Kim et al. (2004); **12**. Kim et al. (2005); **13**. Song et al. (2006); **14**. Lee et al. (2011b); **15**. Kida et al. (2009); **16**. Lee et al. (2009); **17**. Zhang et al. (2001); **18**. Wang et al. (2011); **19**. Mcknight et al. (2008b); **20**. Zhang et al. (2011); **21**. Bai et al. (2010); **22**. Nieminen et al. (2011); **23**. Song et al. (2008); **24**. Lee et al. (2011a); **25**. Dong et al. (2005).

We identified through exome sequencing and confirmed by bidirectional Sanger sequencing analysis of *DSPP*, a heterozygous deletion of 1 base pair (bp) in exon 5 (p.[Ser1226Alafs^*^88];[=] or c.[3676delA];[=]) in 9 affected patients (Figure 3). Segregation analysis validated the absence of the mutation in unaffected individuals. The deletion was absent from dbSNP and the Exome Variant Server. These mutations are predicted to cause a frameshift from codon Ser1226 producing an early stop codon 87 amino acids after the deletion and deleting the protein of an important functional domain.

## Discussion

This work is an extraordinary travel in time, human beliefs and mutual assistance, genetics, science, and new technologies allowing the understanding of the exceptional prevalence of DI in this remote village from Madagascar. The family believed that the tooth coloration and the disease was of nutritional origin. Some hypotheses were proposed like the large consumption of red rice, or drinking habits (acidic water source). Oral tradition of the family history described healthy ancestors. Tradition forbids women, after giving birth, to eat white rice and transgression of this law after a famine period was believed to be associated with the appearance of the dental defect among the population.

DI incidence is believed to reach 1 in 8000 individuals according to Barron et al. (2008). Due to the founder effect, well observed in generation I of the pedigree, and the geographic isolation of the studied population, this prevalence approximates 40% in this population.

DI is transmitted as an autosomal dominant trait and this is clearly visible with the parent to child transmission seen in the pedigree and the presence of affected members in each generation.

The medical history revealed hearing loss problems, which indeed have been reported as associated with DI and *DSPP* mutations (Xiao et al., 2001). Blue sclerae are a classical hallmark of osteogenesis imperfecta clinical synopsis and the association of DI with even a milder form of osteogenesis imperfecta was still a possible diagnosis (Wang et al., 2012).

The phenotype demonstrated both enamel and dentin defects as was previously also reported (Wang et al., 2011). *Dspp* is expressed by odontoblasts and transiently by preameloblasts (Bronckers et al., 1993; Ritchie et al., 1997; Begue-Kirn et al., 1998).

Difficulties throughout the years to sequence the *DSPP* gene, especially the *DPP* region, are due to the high GC rich contents and the number of repeats. As no mutation could be initially detected in this candidate gene and because of disease high frequency within this population we hypothesized that another unidentified gene might be involved. Thus, we used exome sequencing to look for the causative gene. But in fact, we identified a novel single base pair deletion within the end of the fifth *DSPP* exon leading to a premature stop codon. It has never been described in the literature.

Thirty nine mutations in the human *DSPP* gene causing dentin defects have been previously reported (Figure 3). Mutations (mostly substitutions) leading to a DI (DGI) phenotype are located mostly at the 5′ end of *DSPP* and also seem to cluster in exon 2 and around the splice boundaries of exon 3. In exon 5 at the 3′ end of *DSPP*, deletions causing frame shift mutations were responsible for DGI and dentin dysplasia (DD) (Wang et al., 2011). The mutation described herein is also localized at the end of exon 5. This exon codes for DPP (dentin phosphoprotein), which is one of the most abundant extracellular matrix components in dentin (after collagen type I COL1A1, COL1A2). DPP has a role in biomineralization by binding to collagen and calcium and promoting the nucleation and growth of hydroxyapatite crystals (Prasad et al., 2010). The discovered mutation is predicted to cause a frameshift from codon Ser1226 producing an early stop codon 87 amino acids after the deletion, depleting the protein of an important functional domain. This domain is called “Asp/Ser-rich” by UniProt (position 439-1301).

To date, only one other mutation has been identified in the 3′ end of exon 5 (Dong et al., 2005) and consisted of a 36 bp deletion and an 18 bp insertion with a phenotype of DGI type III. Authors reported affected family members with amber tooth discoloration, opalescent appearance, severe attrition of teeth, visible pulp chambers and shell teeth on radiographs differing from the DI phenotype reported in this paper.

Targeted next-generation sequencing technics for orodental disorders (Prasad et al., 2016) prove to be efficient methods to sequence *DSPP* gene allowing further mutations detection and helping providing accurate molecular and clinical diagnosis to rare disease patients. Differential clinical and molecular diagnosis between DI and mild forms of osteogenesis imperfecta presenting with opalescent teeth is important and will orientate patients toward appropriate integrated dental and medical care. These methods, as associated costs decrease, will be transposed from research results to diagnostic molecular findings.

## Author contributions

JM, RWR, SNR, JCR, GR, ROA, RHR, SER, LHR, JAR collected the salivary samples and detailed the patients' phenotype. JM travelled back and forth between France and Madagascar to develop the project and gathered funding. BR, PG tried to sequence DSPP gene using conventional techniques. MH, CS, VG identified the molecular basis of the disease through NGS assays. MH, CS, VG, MCM, SRA, JAR, HD, ABZ analyzed the data and wrote the manuscript. ABZ designed the study and was involved from conception, funding seeking to drafting and critical review of the manuscript. All authors therefore contributed to conception, design, data acquisition, analysis, and interpretation, drafted and critically revised the manuscript. All authors gave final approval and agree to be accountable for all aspects of the work.

## Funding

This work was financed by grants from: the University of Strasbourg, the Hôpitaux Universitaires de Strasbourg (API, 2009-2012, “Development of the oral cavity: from gene to clinical phenotype in Human”), the EU-funded project (ERDF) A27 “Oro-dental manifestations of rare diseases,” supported by the RMT-TMO Offensive Sciences initiative, INTERREG IV Upper Rhine program, a contribution from EAPD and the INTERREG V RARENET program. This study was also supported by the grant ANR-10-LABX-0030-INRT, a French State fund managed by the Agence Nationale de la Recherche under the frame programme Investissements d'Avenir labeled ANR-10-IDEX-0002-02. ABZ is a fellow of University of Strasbourg Institute for Advanced Study (USIAS) and received USIAS Fellowship. Funding, support as well as dental materials were gathered by JM for her travels from the Conseil Départemental de l'Ordre des Chirurgiens Dentistes du Bas-Rhin, Ville de Strasbourg, Conseil Général du Bas-Rhin, Bureau de la Vie Etudiante de l'Université de Strasbourg, Association Amicale des Etudiants en Chirurgie Dentaire de Strasbourg, Henry Schein, GC Europe, Megadental, Laboratoire Unodis Haguenau, Colgate, Pierre Fabre, Alpha Omega Alsace, Laboratoire Flecher Strasbourg, Crédit Mutuel Profession de Santé. A grant was received in Madagascar from Général Randrianazary “Secrétaire d'état à la gendarmerie” in 2012, to cover travel expenses.

## Web resources

The URLs for data presented herein are as follows:

dbSNP, http://www.ncbi.nlm.nih.gov/projects/SNP/
NHLBI Exome Sequencing Project (ESP) Exome Variant
Server, http://evs.gs.washington.edu/EVS/
OMIM, http://www.omim.org/
PolyPhen-2, http://genetics.bwh.harvard.edu/pph2/
RefSeq, http://www.ncbi.nlm.nih.gov/refseq/
SIFT, http://sift.bii.a-star.edu.sg/
VaRank, http://www.lbgi.fr/VaRank
UCSC Genome Browser, http://genome.ucsc.edu

### Conflict of interest statement

The authors declare that the research was conducted in the absence of any commercial or financial relationships that could be construed as a potential conflict of interest.
