# Corrigendum: Detection of a Novel *DSPP* Mutation by NGS in a Population Isolate in Madagascar

**DOI:** 10.3389/fphys.2016.00304

**Published:** 2016-07-26

**Authors:** Agnès Bloch-Zupan, Mathilde Huckert, Corinne Stoetzel, Julia Meyer, Véronique Geoffroy, Rabisoa W. Razafindrakoto, Saholy N. Ralison, Jean-Claude Randrianaivo, Georgette Ralison, Rija O. Andriamasinoro, Rija H. Ramanampamaharana, Solofomanantsoa E. Randrianazary, Louise H. Ralimanana, Béatrice Richard, Philippe Gorry, Marie-Cécile Manière, Jeanne A. Rasoamananjara, Simone Rakoto Alson, Hélène Dollfus

**Affiliations:** ^1^Faculté de Chirurgie Dentaire, Université de StrasbourgStrasbourg, France; ^2^Centre de Référence des Manifestations Odontologiques des Maladies Rares, Hôpitaux Universitaires de Strasbourg, Pôle de Médecine et Chirurgie Bucco-dentaires Hôpital CivilStrasbourg, France; ^3^Centre National de la Recherche Scientifique-UMR7104, Institut de Génétique et de Biologie Moléculaire et Cellulaire, Institut National de la Santé et de la Recherche Médicale U 964, Université de StrasbourgIllkirch, France; ^4^Laboratoire de Génétique Médicale, Faculté de Médecine, Institut National de la Santé et de la Recherche Médicale U 1112, Université de StrasbourgStrasbourg, France; ^5^Institut d'Odonto-Stomatologie Tropicale de Madagascar, Université de MahajangaMahajanga, Madagascar; ^6^UFR Odontologie de Lyon, Université Claude BernardLyon, France; ^7^Research Unit of Theoretical & Applied Economics, GREThA (UMR Centre National de la Recherche Scientifique 5113), Université de Bordeaux PessacPessac, France

**Keywords:** rare disease, dentinogenesis imperfecta, dental anomalies, dentin, mutation, NGS, humans

Reason for Corrigendum:

The authors, Jeanne A. Rasoamananjara and Louise H. Ralimanana were inadvertently missed in the original manuscript and we wish to add their names for contribution to this work. All authors have agreed to this modification.

The authors apologize for this oversight. This error does not change the scientific conclusions of the article in any way.

The original article has been updated.

## Author contributions

ABZ, MH, CS, JM, VG, RWR, SNR, JCR, GR, ROA, RHR, SER, LHR, BR, PG, MCM, JAR, SRA and HD approved the content of Corrigendum.

### Conflict of interest statement

The authors declare that the research was conducted in the absence of any commercial or financial relationships that could be construed as a potential conflict of interest.

